# Effect of operational parameters on droplet deposition characteristics using an unmanned aerial vehicle for banana canopy

**DOI:** 10.3389/fpls.2024.1491397

**Published:** 2025-01-09

**Authors:** Jiaxiang Yu, Xing Xu, Jieli Duan, Yinlong Jiang, Haotian Yuan, Huazimo Liang, Shuaijie Jing, Zhou Yang

**Affiliations:** ^1^ College of Engineering, South China Agricultural University, Guangzhou, China; ^2^ Guangdong Laboratory for Lingnan Modern Agriculture, Guangzhou, China; ^3^ School of Mechanical Engineering, Guangdong Ocean University, Zhanjiang, China

**Keywords:** aerial application, unmanned aerial vehicle, banana, droplet deposition, operational parameters

## Abstract

In recent years, as an important part of precision agricultural aviation, the plant protection unmanned aerial vehicle (UAV) has been widely studied and applied worldwide, especially in East Asia. Banana, as a typical large broad-leaved crop, has high requirements for pests and diseases control. The mechanization degree of plant protection management in banana orchard is low. Therefore, our study focuses on the effects of different flight heights (3-5 m) and droplet sizes (50-150 μm) of plant protection UAV on the droplet deposition distribution characteristics of banana canopy. And the droplet deposition distribution in banana canopy and spraying drift of plant protection UAV and ground air-assisted sprayer were compared. The results showed that droplet size was the main factor affecting droplet deposition density, coverage, uniformity and penetration on both sides of banana canopy leaves. The droplet deposition density and coverage on the adaxial side of leaves were mostly significantly larger than that on the abaxial side. The flight height of 4 m and the droplet size of 100 μm could make the adaxial side of banana canopy leaves have higher droplet deposition density (63.77 droplets per square cm) and coverage (12.75%), and can make the droplets effectively deposit on the abaxial side of banana canopy leaves, with droplet deposition density of 17.46 droplets per square cm and coverage of 1.24%. Choosing an appropriate flight height and a droplet size could improve the droplet deposition uniformity on both sides of banana canopy leaves, but the improvement was not significant. Moreover, at a same operational parameter combination, it was difficult to achieve the best droplet deposition density, coverage, uniformity and penetration at the same time. In addition, appropriately increasing the flight height and droplet size could help to improve the droplet deposition penetration on the adaxial side of banana canopy leaves, but there were few significant improvements. Compared with the plant protection UAV, the ground air-assisted sprayer had higher droplet deposition density and coverage on the abaxial side of banana canopy leaves, but had smaller droplet deposition coverage on the adaxial side. The droplet deposition density and coverage on the abaxial side of banana canopy leaves were obviously larger than the adaxial side during the spraying of ground air-assisted sprayer. The droplet drift distance of the ground air-assisted sprayer was farther than the plant protection UAV. The test results of this study can provide practical and data support for the UAV aerial application in banana orchard, and provide a valuable reference for the implementation of air-ground cooperation spraying strategy in banana orchard, which is of great significance to promote sustainable and intelligent phytoprotection of banana orchard.

## 1 Introduction

The banana (Musa spp.), is the most traded and consumed fruit in the world, mainly planted in tropical and subtropical area, and it is an important economic crop and food crop (Guo et al., 2020; Xie, 2019). The banana industry, as the industry with the largest output value of single crop in the tropical region China, is very important for increasing farmers’ income, agricultural efficiency and promoting rural development and revitalization (Jiang T. et al., 2023). As a typical large broad-leaved crop, banana has the characteristics of closed canopy and interlaced branches and leaves, and has high requirements for pests and diseases control (Jiang et al., 2022; Jiang Y. et al., 2023; Zhang et al., 2021). Spraying chemical pesticides to control pests and diseases is one of the important links in the production and management of the banana orchard which directly affects the yield and fruit quality of bananas, and is crucial to the economic benefits of the entire banana industry (Jiang T. et al., 2023; Robson et al., 2007). Therefore, it is of great significance to enhance the effective chemical control of banana pests and diseases for the development of banana industry.

Banana orchards in China are mainly distributed in hilly and mountainous areas such as Guangdong, Guangxi and Fujian (Xu and Liu, 2021). Due to the lack of special pesticide spraying equipment for banana orchard, traditional ground spraying equipment such as self-propelled sprayers or knapsack sprayers are usually used to improve pesticide deposition by over-spraying method, resulting in serious pesticide residues and ecological environment pollution (Barraza et al., 2020; Sinha, 2020). The use of self-propelled sprayer or artificial knapsack sprayer in banana orchard requires the construction of roads where the land is sufficiently flat for the sprayer and the operator to pass through, which will greatly increase the cost and reduce the banana planting area (Matthews, 2009). In addition, where the self-propelled sprayer is used to spray bananas the nozzles need to be elevated above the banana canopy in order for droplets to be dispersed horizontally across the canopy to reach the young leaves. When using the knapsack sprayer, the lower banana leaves will prevent the droplets from reaching the leaves at the top of the canopy. At present, these problems have seriously affected the economic benefits of banana industry in China, and it is necessary to apply more efficient and safe pesticide spraying technology and equipment to banana orchard management.

In recent years, plant protection unmanned aerial vehicle (UAV) has gradually become the preferred plant protection equipment in China due to its special advantages over traditional pesticide spraying equipment (Lan and Chen, 2018). The rotor downwash airflow of the plant protection UAV can disturb the crop leaves, so that droplets can be attached on both sides of the leaves. In addition, the operation of UAV is not limited by terrain and crop growth, and does not destroy the soil structure, while the operator has no risk of poisoning (Xue et al., 2008; Zhou et al., 2014). Therefore, using the plant protection UAV to spray pesticides in banana orchards is an important method to improve the utilization rate of pesticides and achieve high deposition of pesticides on banana leaves. It is also an important way to solve the bottleneck of pesticide application in the banana industry.

With the rapid development of China’s agricultural aviation, the spraying technology of plant protection UAV has become a research hotspot (Chen et al., 2020). Due to the application of plant protection UAV in orchards is still in the initial stage, there are still a series of serious problems in UAV spraying technology, such as unclear optimal operational parameters, poor droplet penetration, low droplet coverage, and uneven droplet distribution (Qin et al., 2016). Aiming at these problems, some researchers have conducted a series of field spray researches on the effects of plant protection UAV operational parameters on the droplet deposition distribution characteristics of fruit crop, such as areca (Wang et al., 2019b; Wang J. et al., 2020; Wang et al., 2023), peach (Meng et al., 2020), citrus (Chen et al., 2017b; Nascimento and Vitória, 2022; Tang et al., 2018), apple (Li et al., 2018; Liu et al., 2020), pineapple (Wang et al., 2019a), grape (Biglia et al., 2022), guava (Verma et al., 2022), coconut (Pandiselvam et al., 2024). These researches mainly focus on forest fruit crops with spindle-shaped canopy, with a few studies on broad-leaved fruit crops, only large broad-leaved woody crops such as coconut and areca, but the research on large broad-leaved herb crops such as banana is almost zero. The shielding effect between leaves of the forest crop is small, and the droplets wrapped by airflow are easy to enter the canopy through the gap between leaves (Guo X. et al., 2021). However, the canopy of large broad-leaved crops such as banana is inverted triangle shaped, with leaves are similar to cattail fan, resulting in a great shielding effect between the leaves. Different from banana leaves, the leaves of coconut and areca have feathery compound leaf structure, which can reduce the shielding effect between leaves. The difference in crop shape and structure makes it difficult to apply the existing research results to banana pest and disease control. In addition, the existing research on broad-leaved fruit crops does not consider the droplet deposition effect on the abaxial side of crop canopy leaves (Wang et al., 2019b; Wang J. et al., 2020; Wang et al., 2023; Pandiselvam et al., 2024). A large number of research results show that the abaxial side of banana leaves is the main occurrence area of pests and diseases because of its suitable temperature, not being directly washed by rain, and not being directly exposed to sunlight (Huang and Wei, 2017). Therefore, the effective droplet deposition on the abaxial side of banana leaves is of positive significance for crop protection by chemical pesticide spraying. Flight height and droplet size are the main factors affecting the application effect of plant protection UAV (OECD, 2021). Too high flight height may cause a significant decrease in droplet deposition on crop canopy leaves; too low flight height may aggravate the occlusion between the crop canopy leaves, thus influencing the droplet deposition effect on the lower canopy leaves (Chen et al., 2022). The droplet size is not only a key index to characterize the pesticide properties, but also an important factor affecting the droplet deposition distribution in crop canopy (Wang et al., 2015; Yao et al., 2017). The droplets of a small size can easily penetrate the crop canopy to deposit with a high deposition, while the droplets of a large size tend to accumulate, slide or bounce, thus affecting the droplet deposition effect (Ding et al., 2022; Qin et al., 2016).

In summary, the study of droplet deposition distribution of banana canopy by plant protection UAV is crucial for the pests and diseases control of banana orchard. This study aimed to evaluate the effect of droplet deposition on both sides of banana canopy leaves at different flight heights and droplet size parameters. The plant protection UAV operational parameters suitable for banana orchard were selected from the perspective of improving droplet deposition, droplet deposition uniformity and penetration. In addition, the droplet deposition distribution in banana canopy and spraying drift of plant protection UAV and ground air-assisted sprayer were compared. These provide data and practical basis for UAV aerial application in banana orchard, and guide the decision-making of the operation model of mechanized pesticide spraying in banana orchards.

## 2 Materials and methods

### 2.1 Plant protection UAV and its spraying system

**Figure 1:**
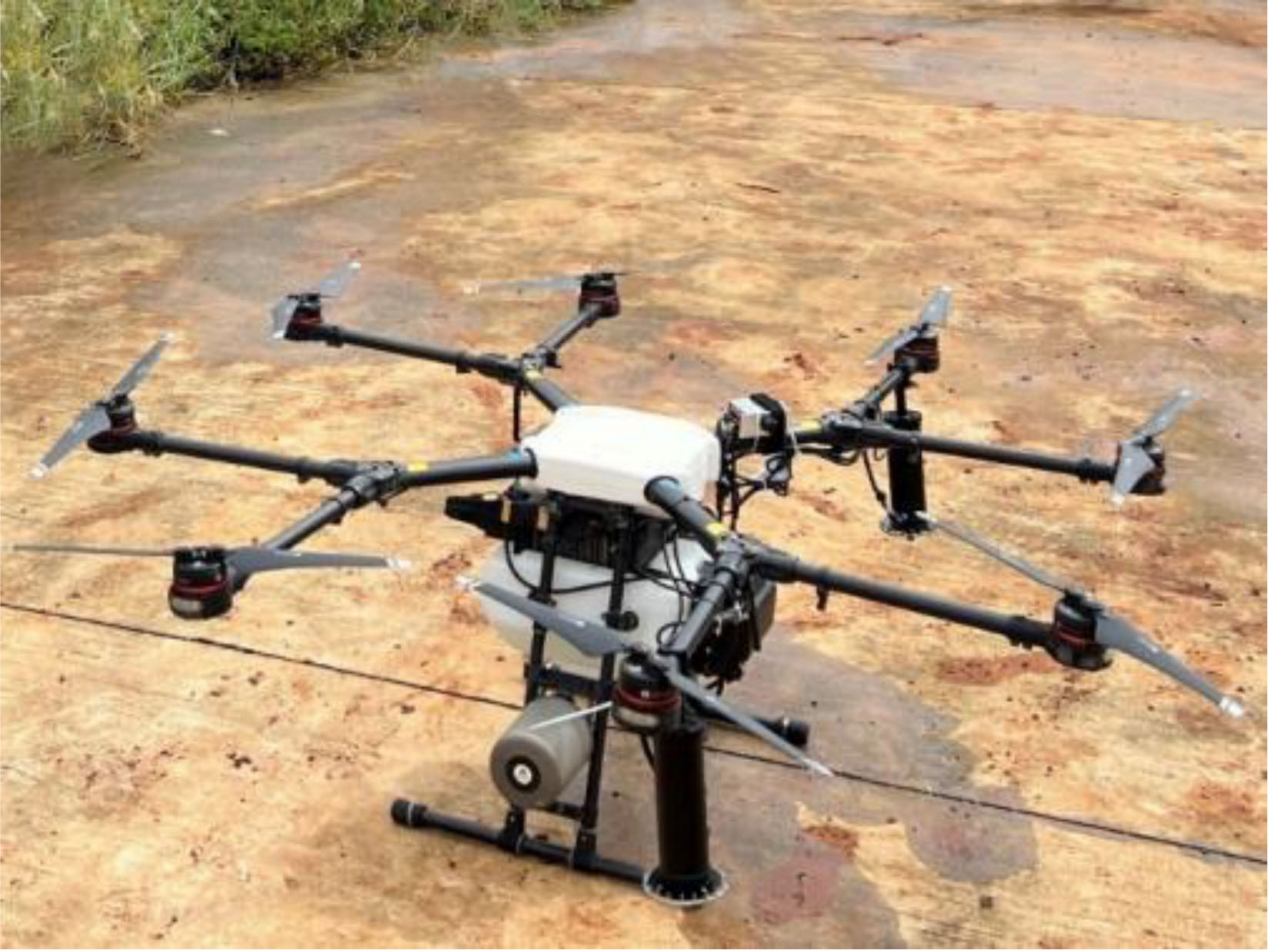
UAV and spraying system used in the test.

As shown in **Figure 1**, the model of UAV used in this spraying test was eight-rotor electric UAV (MG-1P, Shenzhen DJI Technology Co., Ltd., Shenzhen, China). The size of the UAV was 1460 mm × 1460 mm × 616 mm, the rotor diameter was 533 mm, the maximum pesticide tank load was 10 L, the maximum flight speed was 7 m/s, the maximum flight height was 30 m, and the flying time was about 10 min with a full tank. The spraying system included a diaphragm pump and two centrifugal atomizers developed by our team (Yang et al., 2021). The two atomizers were installed under the two main propeller shafts on the rear side of the UAV. The effective spray width was 4-6 m. The atomizers and water pump were both powered by the UAV battery, and the parameters were manually set by the controller before each flight.

### 2.2 Experimental design

#### 2.2.1 Experimental area and crop characteristics

**Figure 2:**
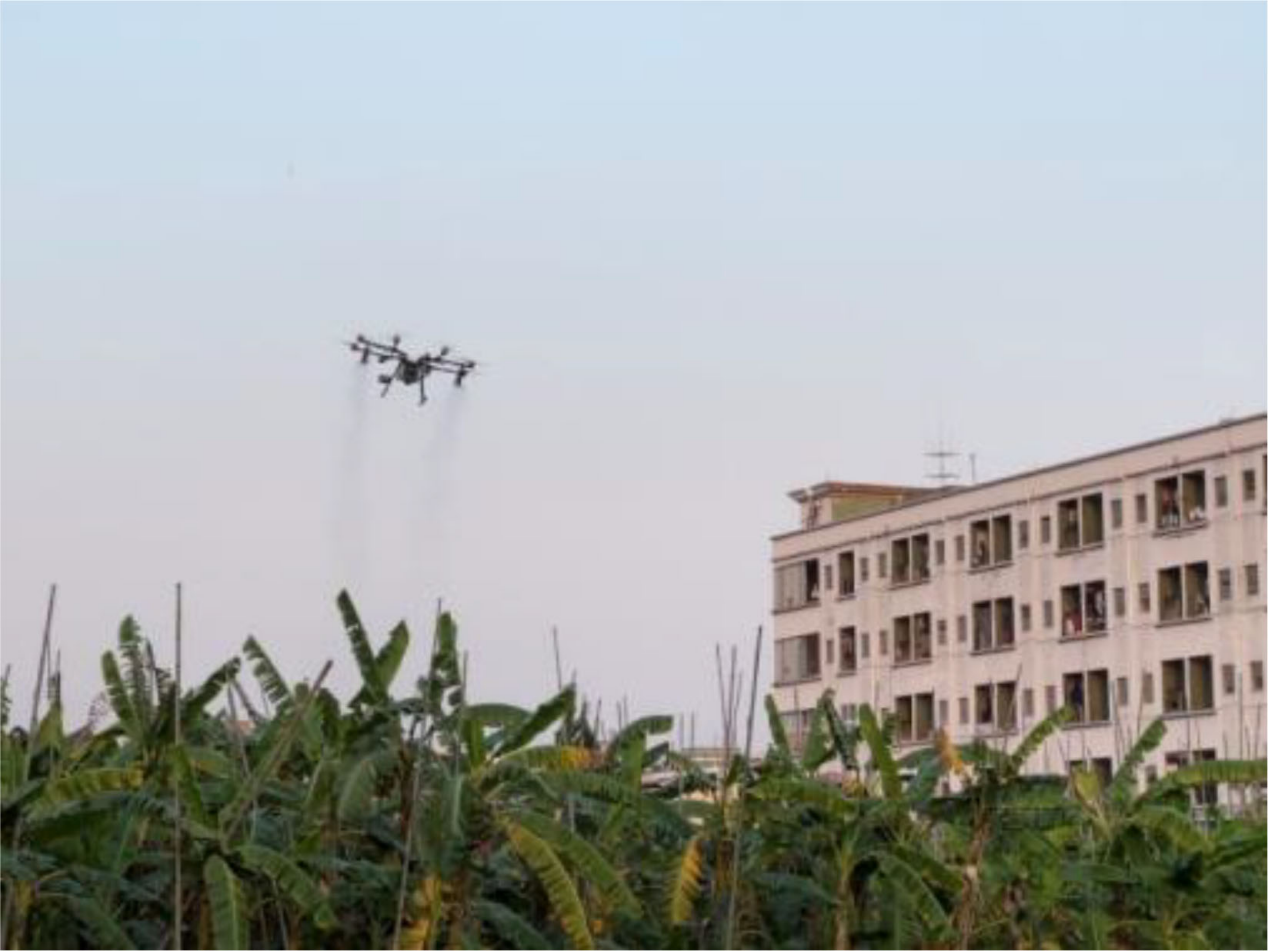
Experimental area.

The tests were conducted on February 23, 2023 at the banana experimental base of the Banana and Vegetable Institute of Dongguan Agricultural Science Research Center located at Dongguan, Guangdong Province, China (latitude 113.698862, longitude 23.006825) (**Figure 2**). The tested crop was Brazil banana in the budding stage. The average height was 3.5 m, and the row spacing was 2.0 m × 2.5 m. Budding stage is an important period in the whole growth stage of banana, which is not only the key to high yield, but also related to the quality of banana (Jing et al., 2022). This stage is also a period of high incidence of pests and diseases, such as leaf spot disease, yellow leaf disease, nematodes, flower thrips, weevil (Constantinides and Jr. McHugh, 2003). Therefore, the budding stage is the key stage of pest and disease management in the whole growth stage of banana.

#### 2.2.2 Sampling scheme

**Figure 3:**
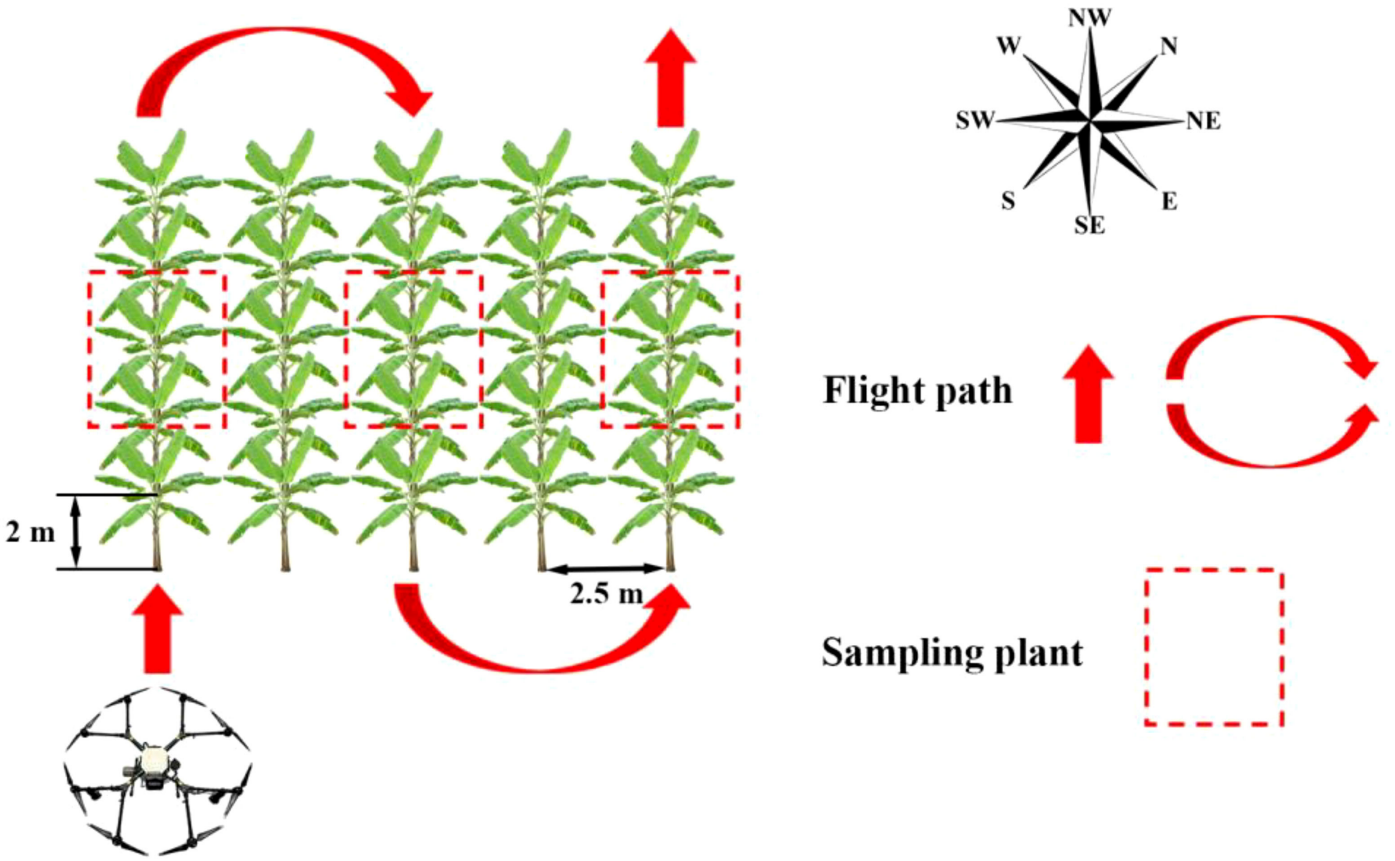
Schematic diagram of site layout and flight path in the test.

In order to reduce the experimental error, the UAV flied along the crop line (northwest - southeast) during spraying test and three healthy banana plants with similar growth condition were selected on the flight path for sampling in each test treatment. The site layout and flight path are shown in **Figure 3**.

**Figure 4:**
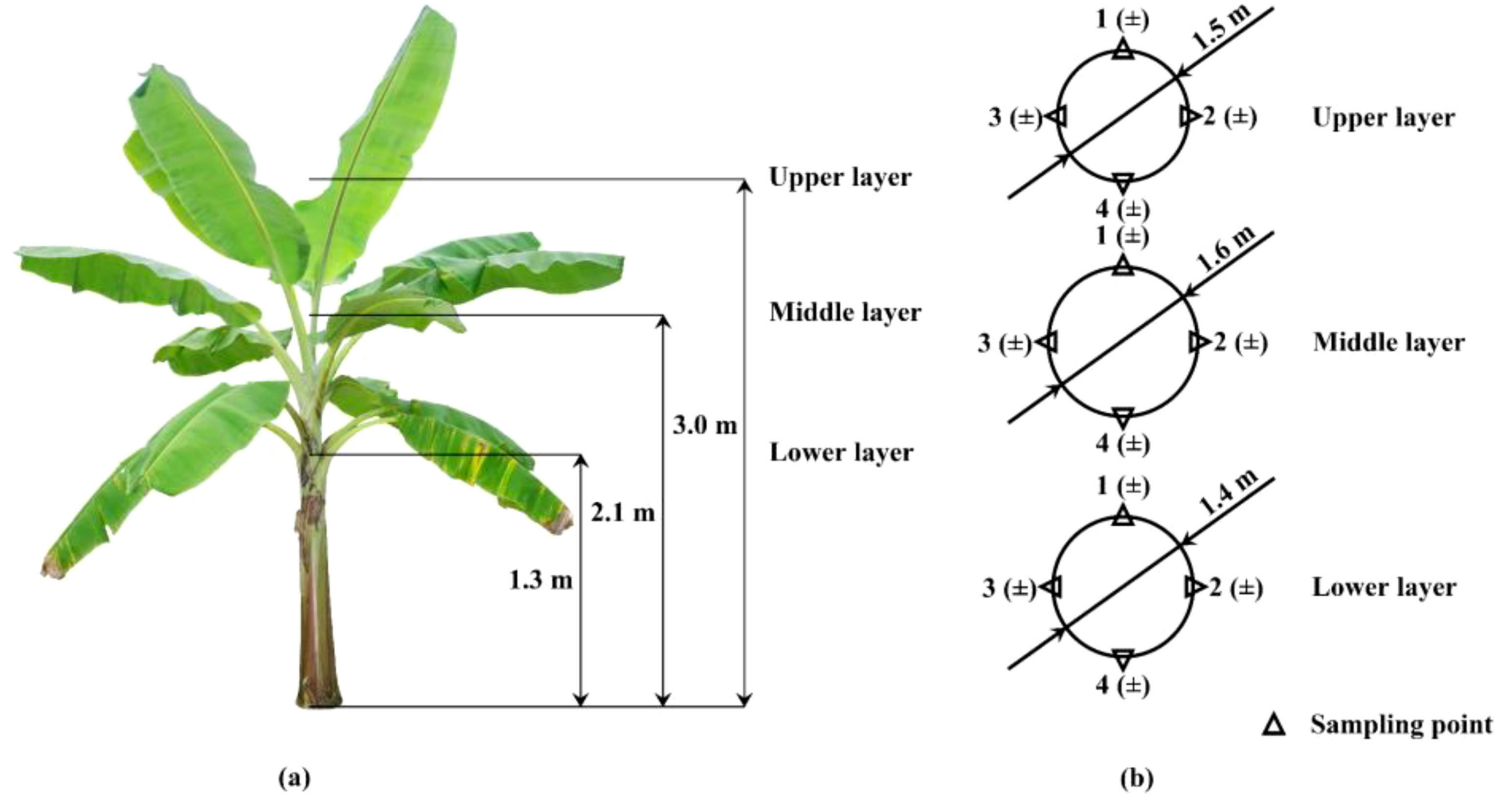
Sampling point layout schematic diagram: **(A)** Canopy sampling layer division; **(B)** Each layer sampling point layout.

The water sensitive paper (40 mm × 30 mm, Chongqing Liuliushanxia Co., Ltd., Chongqing, China) was used to evaluate droplet deposition effect at the sampling location. As shown in **Figure 4A**, there were three sampling layers on the canopy in each sample banana plant, which were upper, middle and lower layers, respectively. Three sampling layers were 3.0 m, 2.1 m and 1.3 m above the ground, respectively. The angles between the upper, middle and lower layer leaves and pseudo-stem were 30°, 60° and 120°, respectively. Among them, the transverse area of the upper layer is small, and the angle between the leaves and the pseudo-stem is small, which is the leaf center part that is prone to pests and diseases. The middle and lower layers have larger transverse area, dense leaves and increased angle with pseudo-stem, which are important parts of banana canopy prone to pests and diseases. In each layer, four sampling points were arranged in a circular and equal array around the pseudo-stem and the diameters of the circular array of the upper, middle and lower layers were 1.5 m, 1.6 m and 1.4 m, respectively (**Figure 4B**). Each sampling point was marked with two numbered white filter paper fixed on both sides of the leaf in advance, so as to accurately place and collect the water sensitive paper on both sides of the leaf at each sampling point, thus avoiding variability. A total of 72 water sensitive paper was collected from three banana plants in each test treatment. This configuration of water sensitive paper and filter paper is widely used to evaluate the actual droplet deposition in crop canopy during spraying tests (Braekman et al., 2009; Miranda-Fuentes et al., 2016; Nuyttens et al., 2004; Zhou and He, 2016).

#### 2.2.3 Operational parameters

Combined with the previous spraying experience of the eight-rotor plant protection UAV operator and banana farmers, it was found that the flight height had a great influence on the spraying effect. Furthermore, in our previous pre-test, it was found that when the flight height was 3-5 m from the top of the banana canopy and the droplet size was 50-150 μm, the droplets could be well deposited in the banana canopy, especially on the abaxial side of banana leaves. Therefore, this experiment mainly aimed at changing the flight height and droplet size of the UAV under the same area spraying amount condition. The flight speed was set to 1.5 m/s and the spraying amount was 75 L/ha. The effects of plant protection UAV flight height (3-5 m) and droplet size (50-150 μm) on droplet deposition distribution were studied by a two-factor and three-level full-factor test. The specific factors and parameters are shown in **Table 1**. The droplet volume median diameter (VMD) (Czaczyk, 2012) was selected as a parameter to characterize the droplet size. The value was controlled by the rotation speed of the centrifugal atomizer and measured by the laser particle-size analyzer in the wind-tunnel lab of the National Center for International Collaboration Research on Precision Agricultural Aviation Pesticide Spraying Technology (NPAAC) in South China Agricultural University based on the ASABE S572.3 standard (ASAE, 2020) according to the method described in our previous study (Yang et al., 2023). The measurement result of VMD (Dv50), Dv10 and Dv90 were shown in **Table 2**, where Dvn represents the sum of the volume of the given size and below, accounting for n% of the total volume of the sample. In addition, the 1‰ OP-10 (DOWSIL, Miland, USA) aqueous surfactant solution was used in the experiment, which has similar physical properties to pesticides (Wang G. et al., 2020). Solvents were prepared before each application and fully mixed.

**Table 1:** Factors and parameters of experiment.

Factors	Parameters
Flight height (m)	3, 4, 5
Droplet size (μm)	50, 100, 150

**Table 2:** Results of droplet size measurement.

Droplet size (μm)	Rotation Speed (r/min)	VMD (μm)	Dv0.1 (μm)	Dv0.9 (μm)
50	9800	50.30 ± 0.95	16.30 ± 0.13	100.51 ± 1.71
100	1650	100.08 ± 0.40	36.61 ± 1.71	333.54 ± 2.36
150	600	150.48 ± 0.70	72.96 ± 1.97	353.32 ± 1.31

The measurement result of droplet size in this table represent mean ± standard deviation.

#### 2.2.4 Weather conditions parameters

Weather conditions parameters were monitored and recorded by portable weather station (Kestrel 5500, Nielsen-Kellerma, Minneapolis, USA) over the full duration of the trials, including wind speed, temperature and humidity. The measurement accuracy of the wind speed, temperature and humidity are ±3%, 1°C and 3%, respectively. The weather station was placed 15 m from the experimental area with a recording frequency of 1 Hz.

The temperature and humidity ranged between 17.1°C and 23.7°C, and 39.6% and 69.1%, respectively, while the wind speed recorded in each test treatment was always below 1.5 m/s. The weather was in the optimal conditions for spraying applications according to the definition of the Best Management Practices (TOPPS-Prowadis Project, 2014). In addition, the angle between the wind direction and the flight direction was less than 30°, which met the requirement of ASAE Standards (ASAE, 2018). In all cases, the experimental trials were conducted in “light air” conditions, making the data obtained from the different test treatments broadly comparable (Barua, 2005). The weather data corresponding to each test treatment are shown in **Table 3**.

**Table 3:** Weather conditions parameters during the test.

Test Treatment	Flight height (m)	Droplet size (μm)	Mean Temperature (°C)	Mean Humidity (%)	Mean Wind Speed (m/s)	Mean Wind Direction
1	3	50	23.0	39.6	0.9	NW
2	3	100	18.8	64.3	0.9	ESE
3	3	150	17.1	68.8	0.8	WNW
4	4	50	23.0	40.7	0.4	WNW
5	4	100	19.7	56.5	0.9	NNW
6	4	150	17.4	69.1	0.5	ESE
7	5	50	23.7	39.5	0.7	NW
8	5	100	23.4	41.0	0.9	WNW
9	5	150	17.9	67.3	0.6	ESE

#### 2.2.5 Comparison of droplet deposition and drift with ground air-assisted sprayer

The tests were conducted on November 7, 2024 at the banana orchard of South China Agricultural University. The tested banana plant was Brazil banana in the budding stage, and the growth was similar to that of the banana plant tested at the Banana and Vegetable Institute of Dongguan Agricultural Science Research Center in February 23, 2023.

Two treatments were set up, which were spraying by plant protection UAV and spraying by ground air-assisted sprayer, respectively. In the treatment of spraying by plant protection UAV, the UAV flied along crop line from north to south and the optimized operational parameter of flight height and droplet size were set. The ground air-assisted sprayer developed by our team was selected as the comparison machine type of the treatment of spraying by ground air-assisted sprayer (Xie, 2024). According to the optimal operational parameters of ground air-assisted sprayer for banana leaves obtained in our previous study, the nozzle type of hollow cone nozzle, the spray pressure of 0.5 Mpa and the wind speed of 4 m/s were set, and the spraying mode was inter-row spraying mode (Jiang Y. et al., 2023). Weather parameters of the two treatments are shown in **Table 4**.

**Table 4:** Weather conditions parameters during the treatment of spraying by plant protection UAV and spraying by ground air-assisted sprayer.

Treatment	Mean Temperature (°C)	Mean Humidity (%)	Mean Wind Speed (m/s)	Mean Wind Direction
Plant protection UAV	22.3	56.8	1.1	ENE
Air-assisted sprayer	21.3	60.2	0.9	ESE

**Figure 5:**
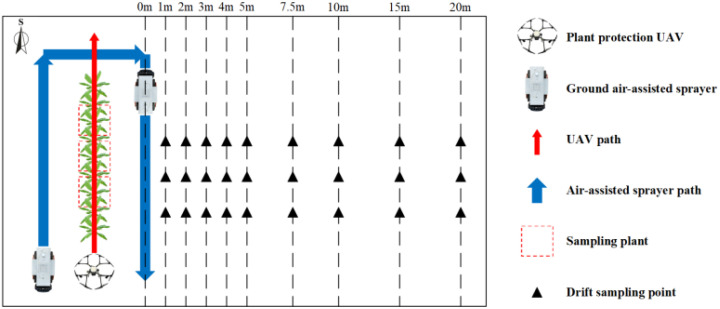
Schematic diagram of site layout and spraying path of plant protection UAV and ground air-assisted sprayer.

The site layout and spraying path of plant protection UAV and ground air-assisted sprayer are shown in **Figure 5**. The drift sampling zone was a bare soil ground without obstacles and vegetation downwind the edge of the sprayed area. Spray drift samples were collected from ground sediment spraying drift. The route of the ground air-assisted sprayer to end the operation was set as the starting edge of the spray drift zone, and a total of 9 drift sampling points were set at 1, 2, 3, 4, 5, 7.5, 10, 15, and 20m away from it. A total of 3 drifting bands were used as 3 replicates, with a total of 27 sampling points per treatment.

### 2.3 Characterization of the deposition effect and statistical analysis

After each test treatment, the water sensitive paper was immediately collected and marked by operators wearing rubber gloves after the droplets had dried, then placed into sealed bags and stored in a cool and dry environment. After all tests, all collected water sensitive paper were taken back to the laboratory and scanned one by one at high resolution (600 dpi) using a printer with an integrated scanner (Hewlett-Packard, Palo Alto, USA). The scanned water sensitive paper was then analyzed by using an open source image processing software ImagePy (Zhang et al., 2024), which can obtain data such as droplet coverage density (the number of water sensitive paper droplets per unit area), droplet deposition coverage (the ratio of the target surface area covered by droplets to the total target surface area) and droplet deposition amount (the deposition amount of droplet per unit area).

According to the existing UAV application standards, the droplet deposition density (15 droplets per square cm) is used to characterize the droplet deposition effect at the sampling position and no indicator for the droplet coverage (Cerruto et al., 2019; Ministry of Agriculture of the PRC, 2018; Fox et al., 2003). At present, there is no evaluation index of canopy effective droplet deposition suitable for large broad-leaved crops. Although droplet deposition density was used as the most important evaluation index when measuring the spray quality of UAVs, it has been proved that percentage area seems to be the most reliable (Salyani and Fox, 1999). In orchard studies, some researchers used droplet deposition coverage of 1% as an evaluation indicator for effective droplet deposition (Wang et al., 2022). Therefore, combined with the previous analysis, this study used droplet deposition density of 15 droplets per square cm and droplet deposition coverage of 1% as the evaluation index for effective droplet deposition. And the droplet deposition amount (μl/cm^2^) was used to characterize the deposition of droplet drift sampling points.

The droplet deposition uniformity was characterized by calculating the mean value of the coefficient of variation (CV) of the droplet deposition density and coverage at each layer’s sampling points. The droplet deposition penetration was characterized by calculating the CV of the mean droplet deposition density and coverage of each sampling layer. Usually, the CV is used as a measure of the uniformity of a set of sampling data by calculating the extent of variation of this set of data. The smaller the CV is, the smaller the change range of the representative data is, which means that the more uniform the droplet deposition, the better the penetration (Liao et al., 2015). The CV calculation equation is as follows:


(1) $$\text{CV}=\frac{S}{\bar{X}}\times 100\%$$

(2) $$S=\sqrt{\sum_{i=1}^{n}{\left(X_{i}-\bar{X}\right)}^{2}/\left(n-1\right)}$$

Where *X_i_* is the droplet deposition density (droplets per square cm) or coverage (%) on the i-th water sensitive paper, $\bar{X}$ is the mean value of *X_i_*, *n* is the total number of samples from the water sensitive paper, and *S* is the standard deviation of *X_i_*.

In this study, all statistical analyses were performed using IBM SPSS Statistics 27 software. Multivariate analysis of variance at the significance level of 0.05 was used to obtain the effects of flight height and droplet size on droplet deposition density, coverage, uniformity and penetration on both sides of banana canopy leaves. The differences of different levels of each factor were analyzed and compared using Duncan’s *post hoc* test at a significance level of 0.05.

## 3 Results

### 3.1 Droplet deposition

**Figure 6:**
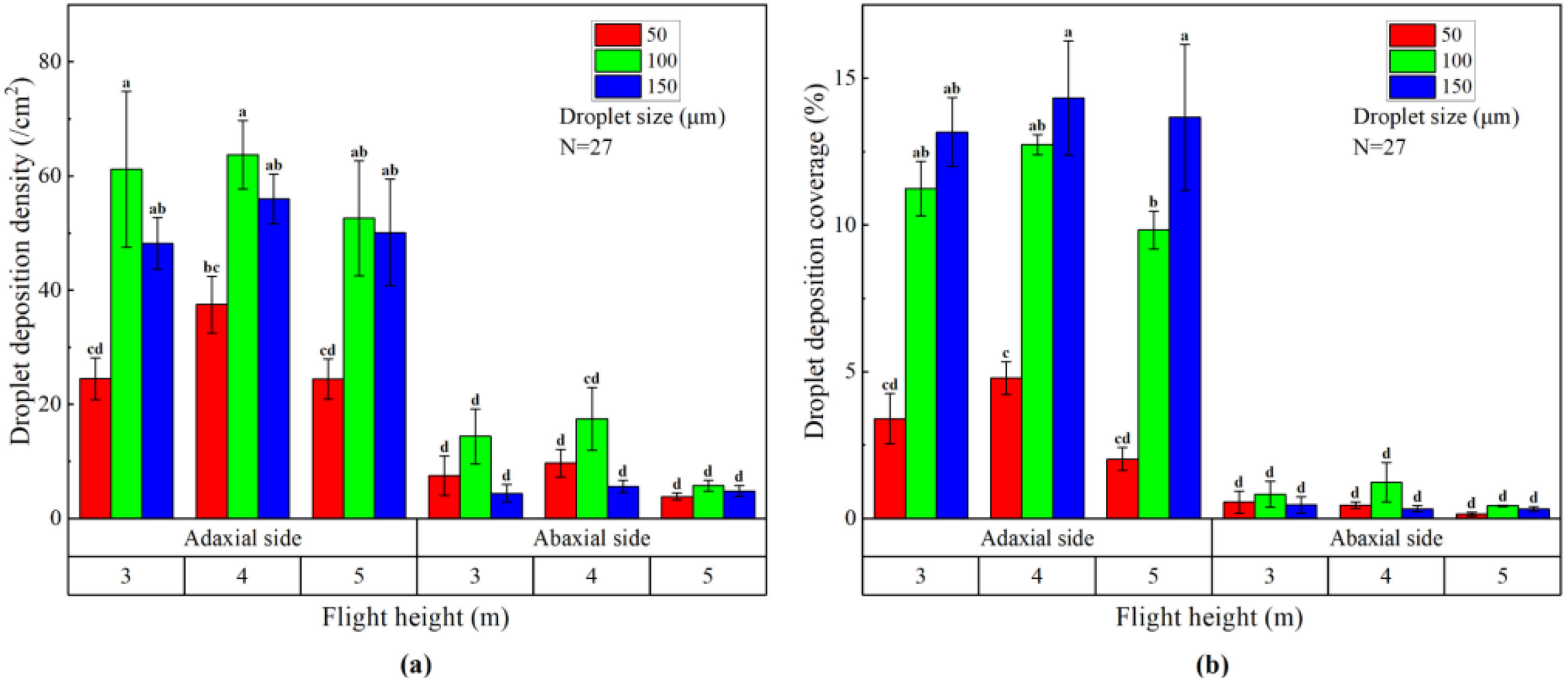
The histogram of droplet deposition density **(A)** and coverage **(B)** on banana canopy leaves. The data in this figure represent mean ± standard deviation. Columns with different lowercase letters are significantly different at the P <0.05 level based on Duncan’s new multiple range test.

The variance analysis results of the effects of flight height, droplet size and their interaction on droplet deposition density and coverage on both sides of banana canopy leaves were carried out, as shown in **Table 5**. The results showed that the droplet size had a significant effect on droplet deposition density on both sides of banana canopy leaves (P < 0.05), and the flight height and their interaction had no significant effect on droplet deposition density (P > 0.05). The droplet size had a significant effect on droplet deposition coverage on the adaxial side of banana canopy leaves (P < 0.05) and had no significant effect on the abaxial side (P > 0.05), and the flight height and their interaction had no significant effect on droplet deposition coverage (P > 0.05). The strongest factor influencing the droplet deposition density and coverage on both sides of the banana canopy leaves was the droplet size, with F values of 8.988, 3.623 and 37.464, 1.344, respectively. The second was the flight height, with F values of 0.998, 2.256 and 1.467, 0.800, respectively. In order to further study the effects of flight height and droplet size on the droplet deposition density and coverage of banana canopy leaves, we conducted a statistical analysis of the test results, as shown in **Figure 6**.

**Table 5:** Variance analysis of droplet deposition density and coverage of banana canopy leaves.

Source	Degree of Freedom	Adaxial Side				Abaxial Side			
		Droplet Deposition Density		Droplet Deposition Coverage		Droplet Deposition Density		Droplet Deposition Coverage	
		F	P	F	P	F	P	F	P
Flight height	2	0.998	0.388	1.467	0.257	2.256	0.134	0.800	0.465
Droplet size	2	8.988	0.002	37.464	< 0.01	3.623	0.048	1.344	0.286
Flight height *Droplet size	4	0.145	0.963	0.202	0.934	0.669	0.622	0.306	0.870
Error	18								
Total	26								

The P and F value indicate the significance level, and P < 0.05 represents factors that have a significant effect on the test result.

It can be observed from **Figure 6** that when the flight height was 4 m and the droplet size was 100 μm, the droplet deposition density on the adaxial side of the banana canopy leaves was the largest (63.77 droplets per square cm). However, the droplet deposition coverage on the adaxial side of 4 m flight height and 150 μm droplet size was the largest (14.33%). When the flight height was 4 m and the droplet size was 100 μm, both the droplet deposition density and coverage on the abaxial side of the banana canopy leaves was the largest, were 17.46 droplets per square cm and 1.24%, respectively.

When the flight height was constant, the droplet deposition density on the adaxial side of the banana canopy leaves increased first and then decreased with increasing droplet size, but the droplet deposition coverage gradually increased with increasing droplet size. However, both the droplet deposition density and coverage on the abaxial side increased first and then decreased with increasing droplet size. But there was no significant difference in droplet deposition density and coverage on the abaxial side between different droplet sizes. Among them, the droplet deposition density and coverage on the adaxial side of 100 μm and 150 μm droplet size was mostly significantly larger than that of 50 μm. When the droplet size was constant, the droplet deposition density and coverage on the adaxial side of the banana canopy leaves increased first and then decreased with increasing flight height, but there was no significant difference in the change. The droplet deposition density on the abaxial side of the banana canopy leaves increased first and then decreased with increasing flight height, however, the droplet deposition coverage on the abaxial side of 50 μm and 150 μm droplet size decreased with increasing flight height, and the droplet deposition coverage 100 μm droplet size increased first and then decreased with increasing flight height. But there was no significant difference in droplet deposition density and coverage on the abaxial side of banana canopy leaves with the change of flight height. In addition, the droplet deposition density and coverage on the adaxial side of banana canopy leaves were mostly significantly larger than that on the abaxial side.

### 3.2 Droplet deposition uniformity

We calculated the droplet deposition density and coverage uniformity of different operational parameter combinations based on the collected droplet deposition data. **Table 6** shows the variance analysis results of the effects of flight height, droplet size and their interaction on the droplet deposition uniformity on both sides of banana canopy leaves. The results showed that the flight height, droplet size and their interaction had no significant effect on the droplet deposition density and coverage uniformity on both sides of banana canopy leaves (P > 0.05). However, the most important factor affecting the droplet deposition density and coverage uniformity on both sides of banana canopy leaves was droplet size, with F values of 2.819, 0.314 and 1.238, 0.461, respectively. The second was the flight height, with F values of 0.172, 0.082 and 0.470, 0.020, respectively.

**Table 6:** Variance analysis of droplet deposition uniformity of banana canopy leaves.

Source	Degree of Freedom	Adaxial Side				Abaxial Side			
		Droplet Deposition Density Uniformity		Droplet Deposition Coverage Uniformity		Droplet Deposition Density Uniformity		Droplet Deposition Coverage Uniformity	
		F	P	F	P	F	P	F	P
Flight height	2	0.172	0.843	0.470	0.633	0.082	0.922	0.020	0.981
Droplet size	2	2.819	0.086	1.238	0.313	0.314	0.734	0.461	0.638
Flight height *Droplet size	4	1.081	0.395	0.746	0.574	1.070	0.400	1.202	0.344
Error	18								
Total	26								

The P and F value indicate the significance level, and P < 0.05 represents factors that have a significant effect on the test result.

**Figure 7:**
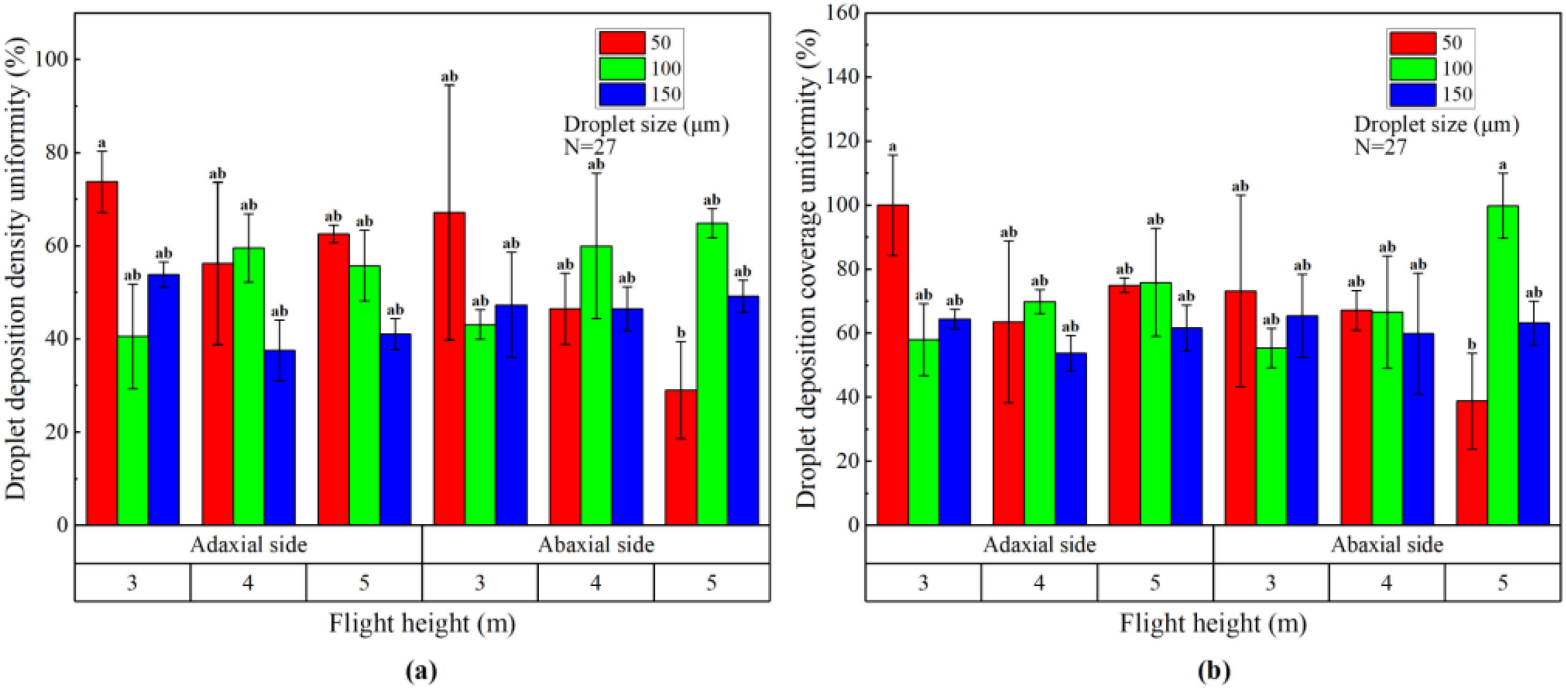
The histogram of droplet deposition density uniformity **(A)** and coverage uniformity **(B)** on banana canopy leaves. The data in this figure represent mean ± standard deviation. Columns with different lowercase letters are significantly different at the P <0.05 level based on Duncan’s new multiple range test.

**Figure 7** shows the droplet deposition density and coverage uniformity on both sides of banana canopy leaves with different operational parameter combinations of flight height and droplet size. It can be seen that when the flight height was 4 m and the droplet size was 150 μm, the droplet deposition density and coverage uniformity on the adaxial side was the best, were 37.58% and 53.79% respectively. When the flight height was 5 m and the droplet size was 50 μm, the droplet deposition density and coverage uniformity on the abaxial side was the best, were 29.06% and 38.83%, respectively. It can be seen that the operational parameter combination that achieved the best droplet deposition uniformity might not be able to achieve the largest droplet deposition density or coverage, and the operational parameter combination that achieved the largest droplet deposition density and coverage mostly had a worse droplet deposition uniformity. We believe that this may be due to the difference in statistical calculation methods, which makes it difficult to balance the optimal droplet deposition density, coverage and uniformity. In addition, when the flight height was constant, there was mostly no significant difference in droplet deposition density and coverage uniformity on both sides of banana canopy leaves with the change of droplet size. When the droplet size was constant, there was also no significant difference in droplet deposition density and coverage uniformity on both sides of banana canopy leaves with the change of flight height. The droplet deposition density and coverage uniformity on the adaxial side of banana canopy leaves was not significantly different from that on the abaxial side.

### 3.3 Droplet deposition penetration

**Table 7** shows the variance analysis results of the effects of flight height, droplet size and their interaction on droplet deposition density and coverage penetration on both sides of banana canopy leaves. The results showed that the flight height, droplet size and their interaction had no significant effect on the droplet deposition density and coverage penetration on both sides of banana canopy leaves (P > 0.05). The most important factor affecting the droplet deposition density and coverage penetration on the adaxial side of banana canopy leaves was droplet size, with F values of 3.142 and 1.504, followed by flight height with F values of 1.025 and 0.450. The most important factor affecting the droplet deposition density and coverage penetration on the abaxial side was the flight height with F values of 2.763 and 0.909, followed by the droplet size with F values of 1.527 and 0.226.

**Table 7:** Variance analysis of droplet deposition penetration of banana canopy leaves.

Source	Degree of Freedom	Adaxial Side				Abaxial Side			
		Droplet Deposition Density Penetration		Droplet Deposition Coverage Penetration		Droplet Deposition Density Penetration		Droplet Deposition Coverage Penetration	
		F	P	F	P	F	P	F	P
Flight height	2	1.025	0.379	0.450	0.645	2.763	0.090	0.909	0.421
Droplet size	2	3.142	0.068	1.504	0.249	1.527	0.244	0.226	0.800
Flight height *Droplet size	4	1.128	0.375	1.585	0.221	1.560	0.228	1.654	0.204
Error	18								
Total	26								

The P and F value indicate the significance level, and P < 0.05 represents factors that have a significant effect on the test result.

**Figure 8:**
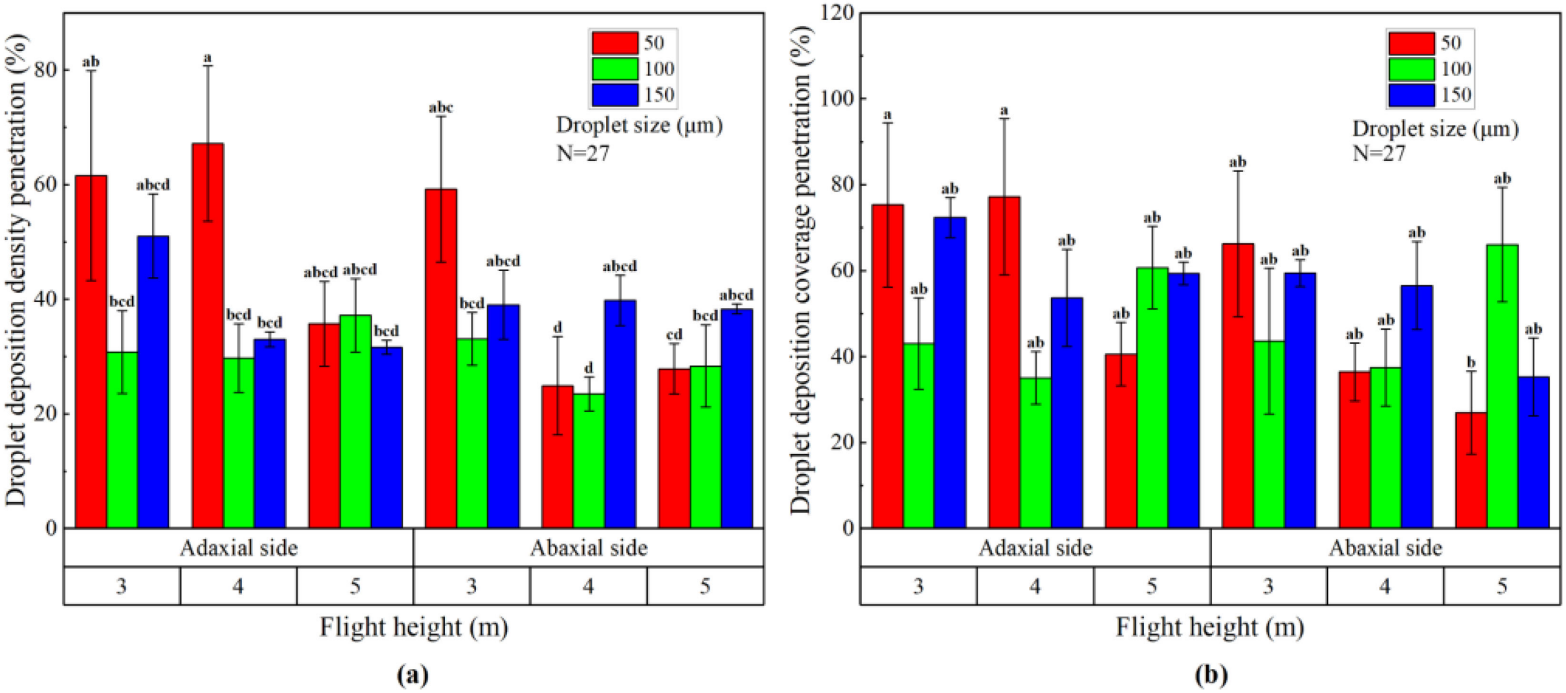
The histogram of droplet deposition density penetration **(A)** and coverage penetration **(B)** on banana canopy leaves. The data in this figure represent mean ± standard deviation. Columns with different lowercase letters are significantly different at the P <0.05 level based on Duncan’s new multiple range test.

**Figure 8** shows the droplet deposition density and coverage penetration on both sides of banana canopy leaves for different flight heights and droplet sizes. It can be seen that when the flight height was 4 m and the droplet size was 100 μm, the droplet deposition density and coverage penetration on the adaxial side of the banana canopy leaves was the best, which were 29.74% and 35.09%, respectively. The droplet deposition density penetration on the abaxial side of 4 m flight height and 100 μm droplet size was the best (23.51%), and the droplet deposition coverage penetration on the abaxial side of 5 m flight height and 50 μm droplet size was the best (26.98%). When the flight height was constant, the difference of droplet deposition density and coverage penetration on both sides of banana canopy leaves was mostly not significant with the change of droplet size. When the droplet size was constant, the difference of droplet deposition density and coverage penetration on both sides of banana canopy leaves was mostly not significant with the change of flight height. In addition, the droplet deposition density and coverage penetration on the adaxial side of banana canopy leaves was mostly not significantly different from that on the abaxial side.

### 3.4 Comparison of plant protection UAV and ground air-assisted sprayer

#### 3.4.1 Droplet deposition

**Table 8** shows the droplet deposition density and droplet deposition coverage on both sides of banana canopy leaves by two kinds of spraying machines, plant protection UAV and ground air-assisted sprayer. It can be seen that the droplet deposition density of the ground air-assisted sprayer on both sides of the banana canopy leaves was 1.6 times and 7.4 times that of the plant protection UAV, respectively. However, the droplet coverage of the ground air-assisted sprayer on the adaxial side of the banana canopy leaf was only 74.6% of that of the plant protection UAV. Different from the plant protection UAV, the droplet deposition density and coverage of the ground air-assisted sprayer on the abaxial side of the banana canopy leaves were obviously larger than the adaxial side.

**Table 8:** The droplet deposition on both sides of banana canopy leaves of plant protection UAV and ground air-assisted sprayer.

Machine Type	Adaxial Side		Abaxial Side	
	Droplet Deposition Density (/cm^2^)	Droplet Deposition Coverage (%)	Droplet Deposition Density (/cm^2^)	Droplet Deposition Coverage (%)
Plant protection UAV	68.21 ± 6.19	17.41 ± 1.97	17.66 ± 2.35	1.31 ± 0.35
Ground air-assisted sprayer	107.23 ± 8.42	12.99 ± 0.81	130.94 ± 3.29	20.69 ± 1.04

The data in this table represent mean ± standard deviation.

#### 3.4.2 Droplet drift

**Figure 9:**
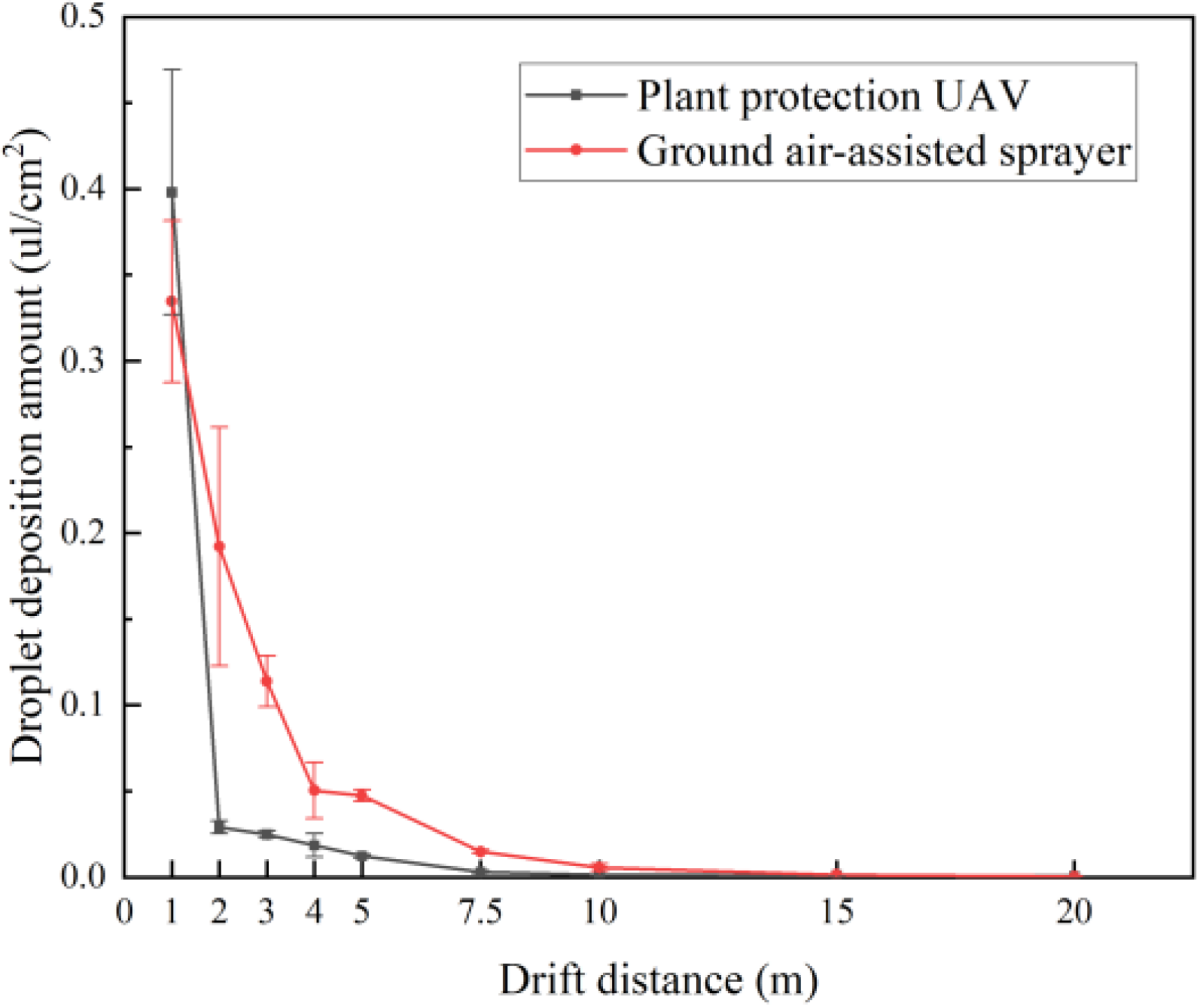
Droplet drift deposition of plant protection UAV and ground air-assisted sprayer.

The ground droplet drift deposition of plant protection UAV and ground air-assisted sprayer within 20 m of the drift zone is shown in **Figure 9**. It can be seen that the droplet drift deposition amount of the plant protection UAV was only larger than that of the ground air-assisted sprayer before 2 m. The droplet drift deposition amount of the plant protection UAV and the ground air-assisted sprayer approaches 0 at 7.5 m and 15 m, respectively.

## 4 Discussion

The results of droplet deposition density showed that neither too large or too small droplet size showed a good droplet deposition density on both sides of banana canopy leaves. Smaller droplets are more likely to drift affected by environmental wind, while larger droplets are less affected by environmental wind and can reach the adaxial side of crop leaves under the action of self-weight, but difficult to be transported to the abaxial side by UAV downwash airflow (Chen et al., 2020). At an appropriate flight height, the appropriate downwash airflow generated by the UAV rotor can force the droplets to be well deposited (Tang et al., 2018). When the flight height was 4 m and the droplet size was 100 μm, the droplet deposition density on both sides of the banana canopy leaves was the largest, and only this operational parameter combination could meet the effective droplet deposition density index of 15 droplets per square cm on the abaxial side.

Different from the results of droplet deposition density, when the flight height was constant, the droplet deposition coverage on the adaxial side of banana canopy leaves increased with droplet size increasing from 100 μm to 150 μm. The reason is that when the application amount is constant, as the droplet size decreases, the number of droplets can increase at a geometric speed, and the probability of droplets hitting the surface of the target crop increases, further increasing the deposition density of droplets on the surface of the target crop. However, the possibility of drifting affected by airflow becomes larger, and the decrease in droplet volume, resulting in a decrease in deposition coverage (Lan et al., 2016). In addition, the droplet deposition coverage on the adaxial side of 4 m flight height and 150 μm droplet size was the largest, but its droplet deposition coverage on the abaxial side did not reach the effective droplet deposition coverage index of 1%. When the flight height was 4 m and the droplet size was 100 μm, the droplet deposition coverage on abaxial side of the banana canopy leaves was the largest and its droplet deposition coverage on the adaxial side was second only to 4 m flight height and 150 μm droplet size. Moreover, only this operational parameter combination could meet the effective droplet deposition coverage index on the abaxial side. Therefore, combined with the results of droplet deposition density we believe that the flight height of 4 m and the droplet size of 100 μm are the best operational parameters for UAV spraying in banana orchard.

The droplet deposition density and coverage on the adaxial side of banana canopy leaves was mostly significantly larger than that on the abaxial side. We believe that this is due to the special spraying method of aerial spraying makes the adaxial side of banana canopy leaves can directly collide with the spray droplets. However, the droplet deposition on the abaxial side always depends on the forced disturbance of the UAV downwash airflow to the leaves, so as to optimize the droplet transport channel and enhance the droplet’s orbital movement ability to indirectly realize the interaction between the droplets and the leaves. This is also the biggest difference between UAV spray and traditional ground spraying equipment (Jiang Y. et al., 2023). In addition, the large banana leaves will be elastically deformed after being subjected to the strong wind load of the UAV downwash airflow, and the leaves on both sides of the veins tend to be parallel to the flow direction of the droplets, resulting in a decrease in the contact area. This is a typical feature of large broad-leaved herbaceous crops such as banana, and it is also the biggest difference from woody plants such as citrus and peach.

Combined with the results of droplet deposition uniformity and penetration, it can be seen that under the same operational parameters, it is difficult to achieve the best droplet deposition, droplet deposition uniformity and penetration at the same time. The droplet deposition reflects the amount of droplets received by the target crop, and the high droplet deposition indicates that the target crop accepts a large number of droplets. The droplet deposition uniformity and penetration reflect the difference of droplet distribution in the horizontal and vertical spraying area, and a large droplet deposition does not mean a good droplet deposition uniformity or penetration. In addition, during the UAV spraying, the deformation degree and inclination degree of leaves at different sampling points was different under the influence of UAV downwash airflow. We believe that this is also a factor that leads to the inconsistency of the optimal operational parameter for droplet deposition, droplet deposition uniformity and penetration. Similar results were found in other herbaceous crops, such as rice (Guo S. et al., 2021).

The results of droplet deposition penetration showed that the droplet deposition density and coverage penetration on both sides of banana canopy leaves at flight height of 3 m was mostly less than that of 4 m and 5 m. When the flight height is low, the wind field under UAV rotor is too strong, causing the upper canopy leaves to swing and tilt around with great deformation. This not only affects the transport of droplets through the upper canopy to the adaxial side of the middle and lower canopy leaves, but also blocks the transport channel of droplets to the abaxial side of the leaves. When the droplet size was 50 μm, the droplet deposition density and coverage penetration on the adaxial side of banana canopy leaves is mostly less than 100 μm and 150 μm. It shows that the penetration ability of larger droplets may be better than that of smaller droplets within a certain range of droplet size for the plant protection UAV. This is because the downwash rotor wind field of plant protection UAV can accelerate the deposition speed of droplets, and blow the upper leaves of crop canopy at the same time, so that a large number of large size droplets with a faster falling speed can reach the lower canopy of the crop within the action time of the rotor wind field (Chen et al., 2017a; Ferguson et al., 2016; Fritz et al., 2009). There is a similar result of the research carried out by some researchers on droplet deposition of different droplet sizes in rice using an UAV (Chen et al., 2020).

Due to the different spraying methods, the ground air-assisted sprayer could obtain larger droplet deposition density and coverage on the abaxial side of banana canopy leaves than the plant protection UAV, but the plant protection UAV had larger droplet deposition coverage on the adaxial side of banana canopy leaves. This also caused the drift droplets of the plant protection UAV to deposit in a large amount in the drift zone near the banana plant, while the droplet drift distance of the ground air-assisted sprayer was farther. Therefore, the application of ground air-assisted sprayers in banana orchards requires a larger drift isolation belt to avoid the harm of pesticide droplet drift to non-target organisms. In addition, the droplet deposition density and coverage on both sides of the banana canopy leaves by the ground air-assisted sprayer were obviously smaller than those of the banana leaves with the petiole fixed on the horizontal beam in our previous study (Jiang Y. et al., 2023). We believe that this is due to the airflow generated by the sprayer fan aggravates the mutual occlusion between the canopy leaves.

Although the results of this study were of great significance for the use of the plant protection UAV in the droplet deposition distribution characteristics on banana plants, the banana morphological structure varies with growth stages and varieties. However, the crop canopy structure directly affects the setting of operational parameters of plant protection UAV (Leandro Da Vitória et al., 2022; Liu et al., 2022; Mahmud et al., 2023; Sun et al., 2022). Further studies are needed to clarify new operational parameters, such as new spraying amounts, flight models, and flight speeds, in order to increase the UAV aerial application efficiency and effectiveness in pests and diseases control in banana orchard. Meanwhile, the results of this study were also of great significance to the air-ground cooperation spraying of plant protection UAV and ground air-assisted sprayer in banana orchards. The two machines work together successively, and the spraying process does not interfere with each other, giving full play to the excellent deposition performance of the ground air-assisted sprayer on the abaxial side of the banana canopy leaves, and then the plant protection UAV supplements the disadvantage of insufficient deposition on the adaxial side of the ground air-assisted sprayer to achieve uniform coverage of banana canopy plant protection operations. This is also our future research focus.

## 5 Conclusions

In this work, aerial spraying tests at different flight heights and droplet sizes were carried out in the banana orchard using an UAV, and the droplet deposition distribution on both sides of banana canopy leaves was studied and analyzed. The main conclusions are shown as follows.

1. The droplet deposition density, coverage, uniformity and penetration of banana canopy leaves were affected by the flight height and droplet size, and the droplet size was the main influencing factor. In addition, different from the ground spraying equipment, the droplet deposition density and coverage on the adaxial side of banana canopy leaves was generally significantly larger than that on the abaxial side during the UAV spraying.
2. The operational parameter combination to achieve the best droplet deposition density and penetration on both sides of banana canopy leaves was the flight height of 4 m, the droplet size of 100 μm, the droplet deposition density and penetration were 63.77 droplets per square cm, 17.46 droplets per square cm and 29.74%, 23.51%, respectively. However, the droplet deposition density uniformity on both sides was worse, which were 59.56% and 60.01% respectively. This operational parameter combination could meet the effective droplet deposition density index of 15 droplets per square cm on the abaxial side of banana canopy leaves. The operational parameter combinations achieved the best droplet deposition density uniformity on both sides of the banana canopy were the flight height of 4 m, the droplet size of 150 μm and the flight height of 5 m, the droplet size of 50 μm, respectively. The best droplet deposition uniformity were 37.58% and 29.06%, respectively. However, the droplet deposition density and penetration on both sides were worse, which were 56.04 droplets per square cm, 24.43 droplets per square cm and 33.02%, 27.91% on the adaxial side, and 5.64 droplets per square cm, 3.86 droplets per square cm and 35.76%, 39.86% on the abaxial side, respectively. Using these two optimal parameter combinations, the droplets could not be effectively deposited on the abaxial side of the banana canopy leaves.
3. Using droplet deposition coverage as an evaluation indicator. The operational parameter combinations achieved the best droplet deposition coverage, uniformity and penetration on the adaxial side of the banana canopy were 4 m flight height and 150 μm droplet size, 4 m flight height and 150 μm droplet size, and 4 m flight height and 100 μm droplet size, were 14.33%, 53.79% and 35.09%, respectively. However, these operational parameter combinations could not reach the best result on the abaxial side, were 0.33%, 59.93%, 37.49%. On the abaxial side, the operational parameter combinations achieved the best droplet deposition coverage, uniformity and penetration were 4 m flight height and 100 μm droplet size, 5 m flight height and 50 μm droplet size, 5 m flight height and 50 μm droplet size, were 1.24%, 38.83%, 26.98%. However, these operational parameter combinations could not reach the best result on the adaxial side, were 12.75%, 75.00%, 40.58%. Only the operational parameter combination of 4 m flight height and 100 μm droplet size could reach the effective droplet deposition coverage index of 1% on the abaxial side.
4. Compared with the plant protection UAV, the ground air-assisted sprayer had higher droplet deposition density and coverage on the abaxial side of banana canopy leaves. However, the droplet deposition coverage on the adaxial side was smaller. The droplet deposition density and coverage on the abaxial side of banana canopy leaves were obviously greater than the adaxial side during the spraying of ground air-assisted sprayer. In addition, the droplet drift distance of the ground air-assisted sprayer was farther than the plant protection UAV.

According to the effective deposition evaluation index of 15 droplets per square cm droplet deposition density and 1% droplet deposition coverage, we believe that the operational parameter combination of flight height of 4 m and droplet size of 100 μm can be used to obtain a better droplet deposition distribution effect on banana canopy leaves without considering the drift risk. Based on the results of this work, our study is still ongoing and further field trials are planned to determine the optimal operational parameters for banana plants at different growth stages. In addition, there were repeated spraying tests in this study, solvent was used instead of pesticide to avoid phytotoxicity. In order to further verify the reliability of plant protection UAV application technology in the banana orchard, it is also necessary to conduct field trials to study the control effect of diseases and pests. And the cooperation spraying strategy of plant protection UAV and ground air-assisted sprayer will be further applied to banana orchards to achieve full-scale droplet coverage of banana canopy. The results of this study provide a valuable reference for the adjustment of the operational parameters of plant protection UAV in the banana orchard and the implementation of air-ground cooperation spraying strategy in banana orchard.

## Data Availability

The raw data supporting the conclusions of this article will be made available by the authors, without undue reservation.
